# Evaluation of work-based screening for early signs of alcohol-related liver disease in hazardous and harmful drinkers: the PrevAIL study

**DOI:** 10.1186/s12889-015-1860-9

**Published:** 2015-06-04

**Authors:** Penny A. Cook, Michela Morleo, David Billington, Kevin Sanderson-Shortt, Colin Jones, Mark Gabbay, Nick Sheron, Mark A. Bellis, Penelope A. Phillips-Howard, Ian T. Gilmore

**Affiliations:** School of Health Sciences, University of Salford, Salford, UK; Centre for Public Health, Liverpool John Moores University, Liverpool, UK; School of Pharmacy and Biomolecular Sciences, Liverpool John Moores University, Liverpool, UK; Greater Manchester Public Health Network, Manchester, UK; Faculty of Health Education and Community, Liverpool John Moores University, Liverpool, UK; Health Services Research, University of Liverpool, and Brownlow Health, Liverpool, UK; Clinical Hepatology, University of Southampton, Southampton, UK; College of Health and Behavioural Sciences, Bangor University, Gwynedd, UK; Department of Clinical Sciences, Liverpool School of Tropical Medicine, Liverpool, UK; University of Liverpool, Liverpool, UK

**Keywords:** Adult, Biomarker, Cirrhosis, Employer, Employee, Fibrosis, Intervention, Non-invasive screening

## Abstract

**Background:**

The direct cost of excessive alcohol consumption to health services is substantial but dwarfed by the cost borne by the workplace as a result of lost productivity. The workplace is also a promising setting for health interventions. The Preventing Alcohol Harm in Liverpool and Knowsley (PrevAIL) project aimed to evaluate a mechanism for detecting the prevalence of alcohol related liver disease using fibrosis biomarkers. Secondary aims were to identify the additive effect of obesity as a risk factor for early liver disease; to assess other impacts of alcohol on work, using a cross-sectional survey.

**Methods:**

Participants (aged 36-55y) from 13 workplaces participated (March 2011–April 2012). BMI, waist circumference, blood pressure and self-reported alcohol consumption in the previous week was recorded. Those consuming more than the accepted UK threshold (men: >21 units; female: >14 units alcohol) provided a 20 ml venous blood sample for a biomarker test (Southampton Traffic Light Test) and completed an alcohol questionnaire (incorporating the Severity of Alcohol Dependence Questionnaire).

**Results:**

The screening mechanism enrolled 363 individuals (52 % women), 39 % of whom drank above the threshold and participated in the liver screen (*n* = 141, complete data = 124 persons). Workplaces with successful participation were those where employers actively promoted, encouraged and facilitated attendance. Biomarkers detected that 30 % had liver disease (25 %, intermediate; 5 % probable). Liver disease was associated with the frequency of visits to the family physician (*P* = 0.036) and obesity (*P* = 0.052).

**Conclusions:**

The workplace is an important setting for addressing alcohol harm, but there are barriers to voluntary screening that need to be addressed. Early detection and support of cases in the community could avert deaths and save health and social costs. Alcohol and obesity should be addressed simultaneously, because of their known multiplicative effect on liver disease risk, and because employers preferred a general health intervention to one that focused solely on alcohol consumption.

## Background

Globally, 5.9 % of all deaths are attributed to the consumption of alcohol [1]. Alcohol-related liver disease is a major contributor to these deaths [2], and accounts for around half of all alcohol-attributed mortality in England [3]. While Europe as a whole saw falling rates of deaths due to liver disease between 1970–2007, the UK and Finland experienced a five-fold increase over the same time period, linked to increased consumption of alcohol [4]. Alcohol-related liver disease disproportionately affects the working age population, and affected individuals are now younger on average than in previous decades: the most common age category for hospital admission for alcoholic liver disease in England dropped from 55–64 years to 45–54 years (from 1989/90 to 2002/03) [5]. The profile of a typical liver disease patient is one of working age, not that of the past stereotypical ‘alcoholic’ [6]. Employers should be concerned about this escalating alcohol harm, because the workplace bears the brunt of the costs. In the USA, excessive alcohol consumption cost the economy an estimated $223.5 billion in 2006, of which 72 % was from lost productivity (including premature mortality and absenteeism) [7]; in the UK, the total costs of alcohol harm were estimated to be £21billion in 2010 [8], of which 35 % was from lost productivity. In contrast, the direct cost to health services in both countries was lower, at 11 % in the USA [7] and 17 % in the UK [8]. Such economic costs are thought to considerably underestimate the impacts on the workplace, since alcohol is also responsible for work absenteeism and underperformance, although quantification of this is difficult. An Australian study found that, amongst employees who consumed alcohol, 3.5 % acknowledged at least one alcohol-related sick day in the previous three months [9]. A longitudinal study in the UK showed that those who consumed more than the recommended ‘safe’ level at baseline (21 units/168 g of alcohol for men; 14 units/112 g for women in a week) had twice as many days off sick during follow-up [10]. At a population level, a 10 % increase in alcohol consumption in Norway was associated with a 6.2 % increase in absenteeism [11]. In addition, productivity decreases if drinking before work (1.8 % of workers reported this in a national US survey) or during the workday (7.1 %, as previously), as well as working under the influence of alcohol (1.7 %) or with a hangover (9.2 %) [12]. The majority of absences are likely to occur in those drinking at risky levels, however, such persons are not necessarily identifiable as being alcohol dependent (for example, classed as occasional binge drinkers [9] or ‘moderately risky’ drinkers [10]).

Obesity also causes liver disease [13], as well as being a possible consequence of over consumption of alcohol because of its calorific content and effects on appetite [14]. Further, obesity may be inadvertently promoted by the working environment, through sedentary behaviour [15]. Large UK cohort studies show that obesity in combination with alcohol use leads to a particularly high risk of liver disease [13, 16], in a ‘supra-additive’ effect. Although damage to the liver is reversible by lifestyle changes if detected early [17], such detection has historically been difficult. Newer diagnostic tests now make early detection possible, but they are rarely used in community or primary care settings. Previous studies have shown alcohol brief interventions (BI—which typically consist of simple advice), to be moderately successful in workplaces [18, 19]. Simple workplace screening using a liver function test and Alcohol Use Disorder Identification Test—AUDIT—was sufficient to elicit a reduction in drinking that remained apparent 12 months later [20].

No previous study has investigated the effect of using newer diagnostic tests for liver disease in the workplace. The Preventing Alcohol Harm in Liverpool and Knowsley (PrevAIL) project aimed to evaluate a mechanism for detecting the prevalence of alcohol related liver disease amongst risky drinkers using fibrosis biomarkers. Secondary objectives were to: detect the prevalence of early stage liver disease using a non-invasive biomarker test to detect fibrosis; determine the interaction of alcohol with obesity as co-risk factors for liver disease; and assess other impacts of alcohol intake on participants’ work and social interactions.

## Methods

### Subjects and setting

The areas of Liverpool and Knowsley in north west England experience particularly high levels alcohol-specific mortality (Liverpool, men: 29.12 per 100,000, women 13.19; Knowsley, men: 16.59; women: 11.79) compared to England as a whole (males: 14.6; females: 6.8) [21] and were thus selected for study. Two thirds (67 %) of persons in the study area were recorded to be in employment [22]. The sampling frame for screening was adults aged 36–55 years. The peak for diagnosing alcohol related liver disease is 45–54 years [5], as such, to screen for those most at risk we used 55 years as the upper cut-off. The study was part of a bigger study (not reported here) that also recruited via general practice and community events [23].

### Sample size calculations

The prevalence of liver damage among employees drinking above the recommended levels was unknown since we could find no similar studies carried out in the community. However, a prevalence study in France using biomarkers found 3 % presumed fibrosis in all persons regardless of alcohol consumption status [24]. We assumed that up to 10 % of all those drinking at risky levels could show signs of damage, as had previously been assumed for a primary care population screening study, the Alcohol and Liver Disease Detection Study (ALDDeS), which subsequently found a prevalence of 11 % [25]. Thus, we estimated a sample size of 100 would be sufficient to estimate prevalence with a 6 % margin of error and a 95 % confidence interval. Since around a third of the population in this region drink at ‘increasing risk’ (or higher) levels (28 %) [26], we estimated that 300 individuals were required for the initial alcohol consumption screen.

### Recruitment

Between March 2011 and April 2012, screening locations were selected following sensitisation briefings with local employers. Medium to large workplaces (employing >400 persons) were first identified through business networks, health promotion agencies and the Chamber of Commerce. A range of employers were then targeted including white collar professionals, manufacturing, public and private sector, local and multi-national companies. The project was marketed to employees as a free health assessment and was promoted through posters, flyers, emails and internal newsletters. Drop-in clinics or pre-arranged appointments were supplied to suit each workplace, and times/days outside the normal working day were offered to accommodate shift workers. Assessments were conducted in private rooms by a researcher (for questionnaires) and a nurse (for clinical screen).

In total, 37 organisations were approached: 13 agreed to take part, ten declined and we were unable to make meaningful contact with the remaining 14. Of the 13, not all were willing/able to tell us how many employees met the age criteria; of the seven who did, screening participation was 1.8–5.5 % in four large organisations (employing >200 eligible persons) and 18–25 % in three small organisations (<200 employees).

### Screening procedure

#### Alcohol consumption screen

Volunteers reaching the inclusion criteria were first screened using a short structured screening form to assess alcohol consumption in the week prior to the survey (for each type of alcohol, the quantity and frequency consumed), demographic information (gender, ethnicity, age and postcode—to enable allocation of an area-level measure of deprivation), and conditions that affect liver function (Hepatitis B or C, liver disease /cirrhosis and diabetes). Those drinking more than the accepted UK threshold for ‘lower risk’ drinking in the previous week (21 units for men, 14 for women, 1 unit = 8 g pure alcohol) [27] were invited to a liver screen.

#### Clinical and liver screen

Height, weight, waist circumference and blood pressure were taken and recorded on case report forms. A 20 ml venous blood sample was drawn into vials appropriate for a conducting a range of standard biochemical and haematological analyses. Blood samples were transported at ambient temperature to the Department of Pathology at Alder Hey Children’s Foundation Trust (Liverpool), processed and analysed within 6 h of venepuncture. Residual serum samples were stored at −80 °C for up to 3 months.

The non-invasive test of liver fibrosis used was the Southampton Traffic Light (STL) test developed by Sheron et al. [17, 25]; this simple algorithm is designed for ease of use and interpretation in primary care. The STL test comprises two serum biomarkers of fibrosis: hyaluronic acid (HA) and procollagen type III N-terminal peptide (PIIINP), together with the platelet count [17]. HA was measured in frozen serum using an ELISA kit supplied by Elitech (formerly Corgenix) and a platelet numbers were determined as part of a full blood count. Frozen serum samples were sent to the Department of Pathology at Southampton University Hospital Trust for analysis of PIIINP by a radioimmunoassay method using kits supplied by Orion. Using the STL system, the risk of fibrosis in participants was categorised according to the following criteria: HA >30 ng/ml or a PIIINP >5.5 μg/ml = score +1; HA >75 ng/ml = score +2; platelet count <150 × 10^9^/l = score +1 [17]. Those scoring zero were categorised as low risk for liver fibrosis and cirrhosis (green), those scoring +1 were intermediate risk (amber—‘some degree of liver fibrosis possible’) and those scoring of ≥2 were high risk (red—‘liver fibrosis probable, with severe liver fibrosis or cirrhosis a possibility’) [25].

Blood pressure was measured with a manual sphygmomanometer, and categorised as high (systolic ≥ 140 mmHg and/or diastolic ≥ 90 mmHg) or normal/low. Weight was taken with light clothing and shoes removed. Height was measured using a portable stadiometer (Leicester Height Measure, Seca). The heights and weights of participants were used to calculate BMI (kg/m^2^). Waist circumferences were also recorded. Because of the limitations of using BMI or waist circumference independently as measures of obesity (for example, BMI does not account for highly muscular adults), the two were used in combination to categorise risk of health complications relating to obesity, using the categories ‘no increased risk’, ‘increased risk’ and ‘high / very high risk’ of obesity-related problems [28, 29].

#### Alcohol behaviours and social impact survey

Participants were given a detailed questionnaire including a measure of alcohol dependency (Severity for Alcohol Dependence Questionnaire: SADQ) [30]. Mild/moderate dependency was defined as a score of 4 or more on SADQ (no participants were severely dependent). Alcohol-related experiences in the last month were assessed by the question ‘have you experienced any of the following after drinking…’ followed by 34 items, including: sleeping better; feeling confident; injuring themselves or others; visiting a general practitioner or a nurse; driving a car. Items relevant for employees and workplaces were analysed for this paper (missed work/class/lectures after drinking; gone to work/class/lectures; present with a hangover, or being late after drinking; avoided clients/customers; avoided a boss/teacher/tutor). The items were worded as ‘work/class/lectures’ to ensure relevance for students as well as working adults in the wider PrevAIL study. Since the individuals recruited for this study were all in the workplace, we have interpreted this item to predominantly reflect performance at work.

### Ethics

The predominant ethical issues were the sensitivity of the topic of alcohol use in the workplace and the arrangements for feedback to participants. At each point, participants were provided with written and verbal information, given the opportunity to ask questions and informed of their right to withdraw. Confidentiality was stressed throughout. A trained nurse gave participants feedback on their alcohol consumption, body mass index (BMI), waist circumference and blood pressure verbally and in writing (on a feedback sheet). Alcohol leaflets that included information on units in different drink types and risks at different levels of drinking were offered to all participants, and those drinking over the recommended limits were advised of this in writing and verbally immediately after the assessment. The leaflets had been developed by Liverpool NHS Primary Care Trust for use in health services locally. Those participating in the liver screen were provided with individual feedback of their blood test results (sent to them by post). Participants’ family physicians were notified of results of blood tests if the participant had given consent for us to do so. Ethics and research governance approvals were gained from Liverpool John Moores University Ethics Committee, Liverpool PCT, and NHS Ethics (reference number: 10/H1013/65).

### Statistical analysis

Lab and clinical data were merged with alcohol behaviour and impact data using participant identifiers. Data were analysed using SPSS v.17. Where data were available (*n* = 333), individual postcodes were assigned to deprivation quintiles based on Index of Multiple Deprivation (IMD) 2010 Scores. Red (probable fibrosis) and amber (possible fibrosis) STL outcomes were categorised as positive. Pearson χ^2^ was used to explore relationships between STL outcome, demographics and risk factors. Demographics and risk factors were combined into a single logistic regression model to find predictors of a positive STL.

## Results

### Recruitment

A total of 363 individuals from 13 workplaces were recruited to the study, just over half of whom were women and most were white British (97 %; Table 1). According to their previous week’s consumption, 27 (7.4 % of the sample) were higher risk drinkers and 114 were increasing risk drinkers (31.4 %), and these individuals were invited to take part in the liver screen.

**Table 1:** Demographic details of individuals screened for alcohol consumption (n = 363)

	Number	Percentage
Gender		
Male	173	47.7
Female	189	52.1
Missing	1	0.3
Age (years)		
36-45	182	50.1
46–55	181	49.9
Ethnicity		
White British	352	97.0
Other	9	2.5
Missing	2	0.6
Deprivation quintile		
1 (most affluent)	54	14.9
2	59	16.3
3	72	19.8
4	49	13.5
5 (most deprived)	99	27.3
Missing	30	8.3
Drinking classification^a^		
Non drinker	13	3.6
Lower risk or none in last week	209	57.6
Increasing risk	114	31.4
Higher risk	27	7.4

^a^For men, lower risk: <22 units in previous week; increasing risk: 22–50 units; higher risk: >50 units. For women: lower risk: <15 units; increasing risk: 15–35 units; higher risk: >35 units

In total, 141 people were invited and 124 took part. Only one person chose not to participate, with the remaining 16 unable to provide a sufficient sample and are excluded from further analysis. Of the 124 screened individuals, 62.3 % were male. Overall, 35.6 % lived in the two most deprived quintiles in England. Those who were eligible but were unable/unwilling to provide a blood sample had drunk less alcohol in the previous week (median 23 units vs 31 units: *P* = 0.044) and were younger (77 % were aged 36–45 years and 23 % 46–55 years, whereas 49 % of the final screened group was 36–45y: *P* = 0.035).

### Findings from the liver screen

The STL test defined seven (5.6 %, 95 % CI 1.48–9.71 %) participants as high risk of liver disease (red: probable fibrosis). All were ‘increasing risk’ drinkers. STL also defined 30 (24.2 %, 16.7–31.7 %) further participants as intermediate risk for liver disease (amber: possible fibrosis), with the remainder (87, 70.2 %) at low risk. Being STL positive (red or amber) was significantly associated the frequency of general practice (family physician) visits (*P* = 0.036) (Table 2), while there was a tendency for obesity (*P* = 0.052) and deprivation (*P* = 0.077) to increase the risk of liver disease. In logistic regression, demographics, drinking history and metabolic indicators (obesity and blood pressure) were not statistically significant predictors of STL positivity (i.e. liver disease). There was a ~3-fold higher odds of a positive STL result among persons who were mildly or moderately dependent on alcohol compared to those with no dependency (AOR = 2.85; CI 0.92–8.84), although this was not significant (*P* = 0.069).

**Table 2:** Factors associated with a positive liver disease screen using the Southampton Traffic Light classification system (n = 124)

Characteristic	Univariate analysis^a^			Logistic regression		
	n	%	P	AOR	95 % CI	P
Gender			0.181			0.276
Male	76	34.2		1.71	0.65–4.47	
Female	48	22.9		*Reference category*		
Age			0.637			0.857
36–45	61	27.9		*Reference category*		
46–55	63	31.7		1.08	0.46–2.56	
Deprivation quintile			0.077			0.141
Quintiles 1 & 2 (most affluent)	47	27.7		*Reference category*		
Quintile 3	31	41.9		1.75	0.62–4.94	
Quintile 4	19	5.3		0.83	0.01–0.83	
Quintile 5 (most deprived)	24	37.5		1.09	0.32–3.67	
Missing	3	33.3		1.61	0.12–22.33	
Frequency of family physician/ nurse visits in last year			0.036	Not included in model.		
Never	22	18.2				
Less than monthly	89	28.1				
At least monthly	12	58.3				
Missing	1	100.0				
Dependence on Alcohol^b^			0.280			0.069
No dependence	98	27.6		*Reference category*		
Mild or moderate dependence	26	38.5		2.85	0.92–8.84	
Drinking classification^c^			0.475	Not included in model.		
Increasing risk	99	31.3				
Higher risk	25	24.0				
Obesity risk classification			0.052			0.094
No increased risk	61	19.7		*Reference category*		
Increased risk	28	39.3		2.54	0.86–7.46	
High risk or very high risk	35	40.0		2.84	0.99–8.17	
Blood pressure			0.385			0.939
Low or normal blood pressure	84	29.8		*Reference category*		
High blood pressure	36	33.3		0.83	0.30–2.29	
Missing	4	0.0		0.00	0.00–0.00	
Total	124	29.6				

*AOR* Adjusted odds ratios

^a^Pearson χ2 ^b^SADQ score 4–34. ^c^For men, lower risk: <22 units in previous week; increasing risk: 22–50 units; higher risk: >50 units. For women: lower risk: <15 units; increasing risk: 15–35 units; higher risk: >35 units

### Other potential work-related harms

Of those who completed the liver screen (*n* = 124), one had missed work after drinking in the last month; and 32 (26 %) had gone to work in the last month but reported their performance was ‘under par’ e.g. being present with a hangover, being present after drinking, or being late after drinking or they had avoided clients/customers or boss after drinking. There was no association between drinking classification (increasing risk, higher risk) and self-reported likelihood of going to work but performing under par due to alcohol (χ^2^ = 0.627, *P* = 0.428). The most common type of under-par performance was self-reported attendance at with a hangover (*n* = 27). Again, there was no association between drinking classification and likelihood of going to work with a hangover (χ^2^ = 0.091, *P* = 0.763).

## Discussion

### Feasibility of screening in workplaces

In total, 363 persons from 13 workplaces took part in the study, and 39 % of these were identified as having consumed more than the accepted UK upper limits of alcohol in the previous week. Around 30 % of these people who drank at risky levels were found to be at increased risk of clinically significant liver disease. Moreover, the individuals detected in this study to have early/moderate liver disease also were heavier users of the NHS (i.e. had more self-reported visits to the family physician) despite (at that point) being asymptomatic for their liver disease. These findings, derived from a variety of workplace types (ranging from office-based workers to factory employees), suggest a substantial impact of alcohol on the workplace. Whilst liver function blood tests have been conducted in workplaces in Sweden as part of a brief intervention [20], PrevAIL is the first study that we know of to incorporate the use of more accurate fibrosis biomarkers in the workplace.

Despite some challenges in accessing workplaces, of those attending the free health assessment, participation in the full liver screen amongst employees identified as drinking at risky levels was very high, at 99 %. Thus, for this step, response rates were higher than for those achieved in other community settings [23, 25]. The wider PrevAIL study also recruited individuals via a postal survey administered by primary care physicians (family doctors). The results are not presented here, but participation was very low (8.4 %, of whom only 18 % responded to our invitation to the clinical examination, bringing the overall response rate to approximately 1.4 %) [23], suggesting that workplaces were a more efficient way of engaging with the target population. Workplaces that showed successful participation were characterised by active encouragement to take part and flexibility by employers with working arrangements. However, there were challenges to implementing screening. Of 37 workplaces contacted, only thirteen took part. Employers declined for a range of reasons: employees not being able to leave their desks; uncertain economic climate (e.g. going into administration); co-occurrence of similar health-related projects; and perceptions of alcohol as being too sensitive. Some organisations predominantly employed people under the age of 35 years (the lower bound for PrevAIL). Screening participation was relatively low in the large organisations (employing >200 eligible persons), ranging from 1.8–5.5 %, while three smaller organisations (<200 employees) recruited at a higher rate of 18–25 %. This was lower on average than a Swedish study (~15 % of ~6000 staff were recruited), which also collected blood samples in workplaces as part of a brief intervention [20]. Participation rates for an online alcohol intervention in the workplace have also been found to be low and variable between organisations, ranging from 2 % and 12 % in local authorities, to as high as 35 % in a petro-chemical company [31].

Despite our best efforts to emphasise confidentiality, anecdotally we heard that some employees had avoided participation due to fear of disclosure. We did not ascertain whether workplaces had alcohol policies in place, such as a zero alcohol in the workplace policy. If this was the case, some employees would be reluctant to undertake screening. This would need to be considered in new studies exploring the feasibility of such initiatives. Among those who took part in an online alcohol intervention in England [31], 40 % were not confident about the confidentiality. It is difficult to assess this lack of confidence among those who do not take part, and further qualitative research should be carried out to understand this and other potential barriers. For some participants, the opportunity to have a liver screen without the involvement their own doctor was attractive, and 13.5 % (*n* = 21) of our participants chose not to provide their family doctor’s contact details.

### Prevalence of liver disease amongst risky drinkers

In total, 124 people identified as risky drinkers donated a full blood sample for this study. Of these, over 1 in 20 were classed within the ‘red’ category and 1 in 4 were ‘amber’. Previous research in a clinic population demonstrated that STL positive (red and amber) individuals have a significantly increased mortality risk: of 641 patients with suspected liver disease followed for an average of 3.4 years, 16 % of red and 3.4 % of amber patients died from liver disease, while there were no deaths among individuals categorised as green (low risk) [17].

Previous studies have been mostly carried out in clinical settings [17], providing measures of prevalence of populations seeking care, and limiting the understanding of risk within the community. The ALDDeS study used the STL algorithm on a community sample of 10,000 individuals recruited through general practice, and found 11 % red and 40 % amber [25]. ALDDeS used a higher risk population comprising those screening positive on AUDIT (versus PrevAIL’s drinks diary). A study of individuals recruited through a community screening programme in France (using an alternative biomarker combination, known as FibroTest) found a prevalence of 1.5 % for confirmed fibrosis and up to 3 % for presumed fibrosis in all persons regardless of drinking status [24]. Thus our prevalence estimates fall within those for the higher risk ALDDeS population and the lower risk French population.

### Predictors of presumed fibrosis

The literature demonstrates a supra-additive effect of alcohol and obesity on the probability of liver damage [13]. Our relatively small sample size was not sufficient to demonstrate this definitively, showing only a marginal association STL positivity and obesity. Nevertheless, strategies are required that act jointly to reduce alcohol consumption and obesity as this would provide greater potential to reduce liver disease than tackling each issue separately. Further, because of the calorific content of alcohol and the associations between drinking alcohol and consuming food [14], reducing alcohol consumption can aide weight reduction. Employers should be motivated to tackle both issues together, since both alcohol and obesity lead to lost productivity [7, 32, 33].

### Impact of alcohol on the workplace

Alongside a high burden of hidden liver disease, we recorded significant impacts of alcohol on the workplace: we estimate that 9 % of the workforce had performance that was affected by alcohol in the previous month (extrapolated from the 39 % of who were drinking at risky levels and filled out the experiences questionnaire). Although alcohol is widely understood to be very costly in terms of productivity, this is mainly estimated from sickness absence data [7, 33]. The true cost of alcohol to the economy is hard to measure but should include being present at work but with reduced performance.

### Potential strategies for improving engagement with workplaces

We found that workplaces were more likely to engage with the screening if we offered a more holistic health check (since alcohol was deemed too sensitive to be the sole focus). Future interventions could have a more explicit aim to tackle multiple health issues of relevance to the workplace. Interventions that simultaneously address obesity and alcohol, or that focus on improving safety and productivity as well as worker health/wellbeing could be considered. The fact that the frequency of physician/nurse visits was associated with a positive screen should be emphasised to employers as evidence of the impact of alcohol on workplace productivity and worker wellbeing. Gaining access to organisations is time consuming, so once this has been achieved it would be more efficient to roll the intervention out as an annual event.

### Limitations

In terms of assessing the prevalence of liver disease amongst the working population, the two main limitations were firstly: the relatively small sample size, leading to insufficient power to detect statistically significant associations of liver disease with demographic variables; and secondly: the representativeness of the screened group given that many workplaces could not or were not willing to take part, and amongst those that did, participation was around 5 %. Nevertheless, the participation was more successful than recruitment by mail via family physicians.

PrevAIL used a drinks diary to screen for risky drinking (based on the quantity consumed in the previous week). Inclusion in the study was influenced by unusually heavy (or light) drinking. An alternative approach would have been to use a brief screening tool such as AUDIT-C, which asks about typical drinking behaviour. We ruled this out because we hypothesised that our target population would be habitual drinkers who drink more than the accepted UK thresholds, but not necessarily sufficiently to score positive on a brief tool such as AUDIT-C. The consumption diary approach also gives useful information on consumption patterns. With a larger sample, this could be used to elucidate the relationship between total quantity, pattern of drinking, and risk of liver disease.

As with any screening test, when moving from the higher risk secondary care setting to the general population, the prevalence of the targeted health condition is likely to be markedly lower, and this affects the positive predictive value of the test. Sheron et al. [17] modelled the STL under a range of prevalence assumptions. The PPV of a ‘red’ grading could be as low as 30 % in a low prevalence community sample (if the true prevalence was as low as 8 % fibrosis), and 12 % for an amber grade. However, the negative predictive value of a green grading was high (98 %). Usually, a low PPV for a screening test would pose an ethical issue of unnecessary treatment for a condition or unnecessary worry about a condition. For risky drinkers, the treatment (here, a lifestyle intervention to reduce drinking) remains necessary given the range of harms attributed to alcohol and the potential for future liver damage.

We originally intended to measure fasting blood glucose, triglycerides and cholesterol in order to detect metabolic syndrome, and thus account for the known relationship between metabolic syndrome and non-alcoholic fatty liver disease [34], and the synergistic relationship between dietary risk and alcohol as risk factors for liver disease [13]. However, collecting a fasting blood sample proved difficult in the workplace, and only seven blood samples were fasting (5.7 % of blood samples collected where fasting status was recorded). This occurred for a variety of reasons: choosing to take part opportunistically on the day of the study and not being prepared for fasting; the timing of availability / appointment (e.g. in the afternoon) made fasting too difficult; and some employers did not wish employees to be working while hungry. Thus, while we were able to provide a risk score for liver disease, we could not rule out non-alcoholic fatty liver disease as a cause.

## Conclusions

A significant hidden burden of alcohol harm was revealed in a sample of workplaces, making the workplace an important setting to addressing alcohol harm. However, more work is required to develop methods to encourage uptake of voluntary screening, including bringing together multiple health issues (e.g. obesity, smoking and alcohol) to: reduce the burden on the workplace of hosting multiple events; reduce participation fatigue by employees and interventions; and reduce the focus on alcohol, which was perceived as a highly sensitive issue. Such programmes require sustained relationships to be built with employers to decrease the time and costs of approaching workplaces for each new intervention.

To our knowledge, this is the first study to use non-invasive biomarkers to detect liver disease in the work place, and our estimates of liver disease prevalence endorse those found by community surveys in other settings. These individuals with probable liver disease were asymptomatic in terms of liver disease but were already utilising more primary health care resource for general health care. A lifestyle intervention that addresses alcohol use would be sufficient in many cases to prevent further development of disease. Early detection and support for these cases could avert deaths and save considerable health and social costs.

## Abbreviations

ALDDeS: Alcohol and liver disease detection study
AUDIT: Alcohol use disorder identification test
AUDIT-C: Alcohol use disorders identification test-consumption
PrevAIL: Preventing alcohol harm in Liverpool and Knowsley
SADQ: Severity for alcohol dependence questionnaire
STL: Southampton traffic light
